# Antioxidants: Scientific Literature Landscape Analysis

**DOI:** 10.1155/2019/8278454

**Published:** 2019-01-08

**Authors:** Andy Wai Kan Yeung, Nikolay T. Tzvetkov, Osama S. El-Tawil, Simona G. Bungǎu, Mohamed M. Abdel-Daim, Atanas G. Atanasov

**Affiliations:** ^1^Oral and Maxillofacial Radiology, Applied Oral Sciences, Faculty of Dentistry, The University of Hong Kong, Hong Kong; ^2^Institute of Molecular Biology “Roumen Tsanev”, Department of Biochemical Pharmacology and Drug Design, Bulgarian Academy of Sciences, Acad. G. Bonchev Str., Bl. 21, Sofia 1113, Bulgaria; ^3^Pharmaceutical Institute, University of Bonn, An der Immenburg 4, 53121 Bonn, Germany; ^4^Department of Toxicology and Forensic Medicine, Faculty of Veterinary Medicine, Cairo University, Giza, Egypt; ^5^Pharmacy Department, Faculty of Medicine and Pharmacy, University of Oradea, Oradea, Romania; ^6^Department of Pharmacology, Faculty of Veterinary Medicine, Suez Canal University, Ismailia 41522, Egypt; ^7^The Institute of Genetics and Animal Breeding, Polish Academy of Sciences, Jastrzębiec, 05-552 Magdalenka, Poland; ^8^Department of Pharmacognosy, University of Vienna, Vienna, Austria; ^9^GLOBE Program Association (GLOBE-PA), Grandville, MI, USA

## Abstract

Antioxidants are abundant in natural dietary sources, and the consumption of antioxidants has a lot of potential health benefits. However, there has been no literature analysis on this topic to evaluate its scientific impact in terms of citations. This study is aimed at identifying and analysing the antioxidant publications in the existing scientific literature. In this context, a literature search was performed with the Web of Science database. Full records and cited references of the 299,602 identified manuscripts were imported into VOSviewer for bibliometric analysis. Most of the manuscripts were published since 1991. The publications were mainly related to the categories *biochemistry/molecular biology*, *food science technology*, and *pharmacology/pharmacy*. These topics have been prolific since 1990 and before. Polymer science was prolific before, but its publication share declined in the recent two decades. Brazil, China, India, and South Korea have emerged as upcoming major contributors besides USA. Most prolific journals were *Food Chemistry*, *Journal of Agricultural and Food Chemistry*, *Free Radical Biology and Medicine*, and *PLOS One*. Clinical conditions with high citations included Alzheimer's disease, cancer, cardiovascular disease, and Parkinson's disease. Chemical terms and structures with high citations included alpha-tocopherol, anthocyanin, ascorbate, beta-carotene, carotenoid, curcumin, cysteine, flavonoid, flavonol, hydrogen peroxide, kaempferol, *N*-acetylcysteine, nitric oxide, phenolic acid, uric acid, vitamin C, vitamin E, selenium, and resveratrol. Citation patterns temporal analysis revealed a transition of the scientific interest from research focused on antioxidant vitamins and minerals into stronger attention focus on antioxidant phytochemicals (plant secondary metabolites).

## 1. Introduction

Antioxidants existed in many dietary natural sources such as vegetables, fruits, and beverages and dietary antioxidants such as flavonoids may help reduce the risk of mortality from coronary heart disease and incidence of myocardial infarction [1, 2]. Furthermore, epidemiological studies and meta-analyses have suggested that the long-term consumption of plant polyphenols can protect us against a range of diseases, such as cancers, cardiovascular diseases, diabetes, osteoporosis, and neurodegenerative diseases (e.g., Alzheimer's disease) [3–7]. With the aging population and only a small proportion of the population has consumed daily the recommended amount of fruits and vegetables, there are great opportunities in improving the general health and against the degenerative diseases of aging by improving the diet [8, 9].

To show the overall impact of the antioxidant research, a bibliometric analysis of the antioxidant research field may allow a deep understanding of the changes in the field in terms of contributors and hot topics and their citation performance [10–13]. To the best of our knowledge, no such literature analysis has been published for antioxidant research. Therefore, the aim of this study is to identify and analyse the antioxidant-related publications in the existing scientific literature. The primary objectives are as follows:

1. To understand the relevant key research categories in antioxidant research
2. To identify the countries and journals having a major contribution to this research and to evaluate their citation performances during different time periods
3. To reveal which chemicals/pharmaceuticals have had high citation counts during different time periods

## 2. Materials and Methods

### 2.1. Data Source

In May 2018, a literature search was performed with the multidisciplinary Web of Science (WoS) online database (Clarivate Analytics, Philadelphia, PA, USA) to identify papers with the following search strategy: TOPIC = (“antioxida∗” OR “anti-oxida∗”). This strategy searched for papers that contain the word antioxidant/antioxidant and its derivatives in their title, abstract, or keywords. No restrictions were imposed on the publication year, publication type (e.g., original article, review, and editorial), or publication language.

### 2.2. Data Extraction

The manuscripts resulted from the literature search were evaluated and recorded for (1) publication year, (2) journal title, (3) total citation count, (4) authorship, (5) WoS category, and (6) manuscript type. The full records and cited references of these manuscripts were imported into VOSviewer (CWTS, Leiden University, Leiden, The Netherlands) for bibliometric analyses, such as citation performances of institutions, countries/regions, and journals.

VOSviewer extracts and analyses the words in the titles and abstracts of the publications, relates them to citation counts, and finally visualizes the results as a bubble map [14]. Four such bubble maps were generated, one each for 1990 and before, 1991–2000, 2001–2010, and 2011–2018. Each bubble represents a word or a phrase. We excluded the top 5000 common words from the Corpus of Contemporary American English (the list of words was obtained from https://www.wordfrequency.info/free.asp?s=y). Supplementary data sheets in an Excel file (Data File S1) are all the terms remained after the exclusion of the 5000 common words and their citations per publication, per each of the four survey periods. Bubble size indicates the frequency of occurrence of the words (multiple appearances in a single publication count as one). Bubble color indicates the averaged citation count received by publications containing the word in their titles or abstracts. Two bubbles are in closer proximity if the two words had more frequent cooccurrence. The term map visualizes terms that appeared in at least 500 of the included publications during the specific survey period (except for 1990 and before, for which the threshold was set at 10 due to the small number of publications). Similarly, bubble maps were generated to visualize journal performances during the four survey periods. Two bubbles are in closer proximity if the two journals cite each other more frequently. For journal maps, only journals that have published at least 100 papers during the specific survey period were included (except for 1990 and before, for which the threshold was set at 10 due to the small number of publications).

## 3. Results and Discussion

### 3.1. General Results

**Figure 1:**
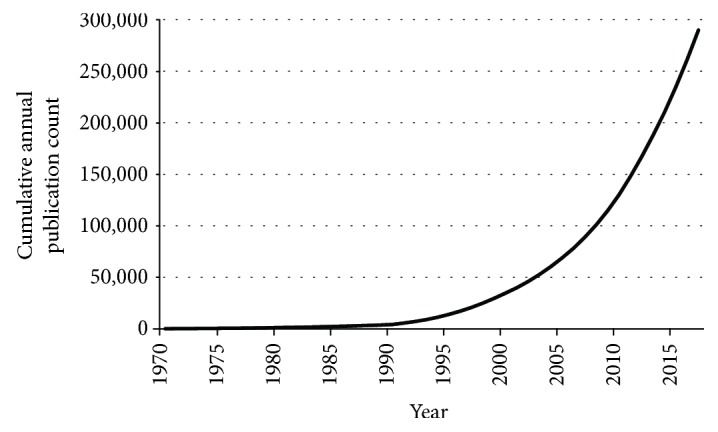
Cumulative annual publication count of antioxidant papers.

There were 299,602 publications identified and analysed. The first publication was published in 1957. It was a publication on studies with antioxidants related to the reduction of serum lipids in humans and rabbits [15]. The first year with more than 100 publications on this topic was 1977, and the first year with more than 1000 publications was 1991. The annual publication trend started to increase steadily since then (Figure 1). Another pivotal point was the year 2007, starting from that year the annual publication count has always exceeded 10,000. In 2017, 28,682 papers related to antioxidants were published. Based on the observed trend, it is reasonable to expect that over 30,000 papers will be published in 2018 alone.

In overall, there were 258,450 (86.3%) original articles and 20,616 (6.9%) reviews in the analysed literature set. The remaining 6.8% of the publications included meeting abstracts, proceedings paper, and editorial material. Most of the publications were written in English (292,778; 97.7%). The publications were mainly classified into the WoS categories of *biochemistry/molecular biology* (45,691; 15.3%), *food science technology* (45,374; 15.1%), and *pharmacology/pharmacy* (35,343; 11.8%). Four most prolific institutions have each accounted for 1% or nearly 1% of total publications, namely, the University of California (4183; 1.4%), Chinese Academy of Sciences (3746; 1.3%), Spanish National Research Council (CSIC, 3483; 1.2%), and Council of Scientific and Industrial Research (CSIR India, 2881; 1.0%). Consistently, USA (52,387; 17.5%), China (39,632; 13.2%), and India (24,958; 8.3%) were the leading countries in publishing antioxidant-related papers. Meanwhile, the most prolific journals that have each accounted for 1% or nearly 1% of total publications were *Food Chemistry* (5818; 1.9%), *Journal of Agricultural and Food Chemistry* (5531; 1.8%), *Free Radical Biology and Medicine* (4332; 1.4%), and *PLOS One* (2854; 1.0%).

In order to perform a systematic analysis and to better evaluate the importance of the research area for the scientific community worldwide, all the 299,602 identified publications were divided into four time periods/decades depending on their publication year, as follows: (i) 1990 and before, (ii) 1991–2000, (iii) 2001–2010, and (iv) 2011–2018.

### 3.2. 1990 and before

There were 4176 publications. In this earliest survey period, the publications were more evenly distributed into different WoS categories, with the five major categories being the *biochemistry/molecular biology* (513; 12.3% of 4176), *food science technology* (507; 12.1%), *polymer science* (414; 9.9%), *pharmacology/pharmacy* (332; 8.0%), and *chemistry, applied* (331, 7.9%). The five most prolific countries/regions were USA (942 publications; 22.6% of 4176; 39.6 citations per publication), USSR (248; 5.9%; 6.2), Japan (165; 4.0%; 35.4), England (145; 3.5%; 51.9), and Canada (90; 2.2%; 69.3). The five most prolific journals were *Journal of the American Oil Chemists' Society* (153; 3.7%; 23.7), *Bulletin of Experimental Biology and Medicine* (123; 2.9%; 0.3), *Federation Proceedings* (87; 2.1%; 3.9), *Abstracts of Papers of the American Chemical Society* (82; 2.0%; 0.01), and *Polymer Degradation and Stability* (82; 2.0%; 11.2).  Figure S1 shows the citation network of the most prolific journals, with existing agglomeration of polymer science journals on the upper right corner, implying that they have cited each other frequently but infrequently cited healthcare and food science journals.

**Figure 2:**
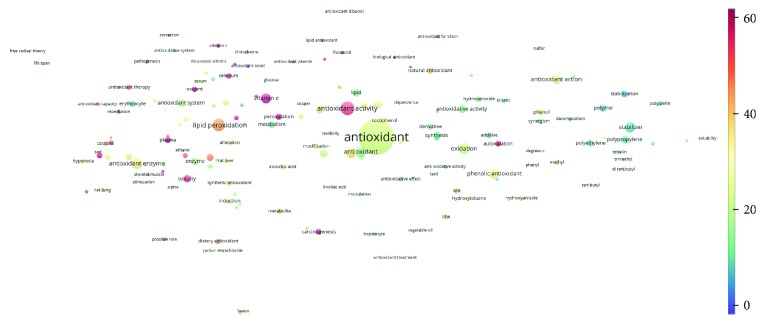
Term map for 1990 or before. The bubble map visualizes 155 terms (excluding the top 5000 common words from the Corpus of Contemporary American English) that appeared in at least 10 of the included publications published in 1990 or before. Bubble size indicates the frequency of occurrence of the words (multiple appearances in a single publication count as one). Bubble color indicates the averaged citation count received by publications containing the word in their titles or abstracts. Two bubbles are in closer proximity if the two words had more frequent cooccurrence.

Topics with high citations included carcinogenesis (45 publications; 71.9 citations per publication) and lipid peroxidation (197; 44.4) (Figure 2). The most cited terms associated with the chemical structures of antioxidants were uric acid (12; 212.1), beta-carotene (12; 189.3), flavonoid (15; 176.5), ascorbate (14; 175.7), vitamin C (22; 69.8), vitamin E (121; 62.7), and selenium (35; 56.3). The chemical structures of all most cited antioxidants or the respective simplest representatives of the related substance classes are illustrated in Table 1.

### 3.3. 1991–2000

There were 30,530 publications. During this period, the WoS category of *biochemistry/molecular biology* has accounted for a larger share of publications (6974; 22.8% of 30,530). The four other major categories were *pharmacology/pharmacy* (3168; 10.4%), *endocrinology/metabolism* (2258; 7.4%), *food science technology* (2245; 7.4%), and *cell biology* (2065; 6.8%). *Polymer science* (547; 1.8%) and *chemistry, applied* (1333; 4.4%) had much reduced share. The five most prolific countries/regions were USA (8837 publications; 28.9% of 30,530; 72.8 citations per publication), Japan (2482; 8.1%; 47.1), Germany (1649; 5.4%; 62.3), England (1534; 5.0%; 76.0), and Italy (1499; 4.9%; 57.9). Russia (570; 1.9%; 13.5) has much reduced share (as compared to USSR in the previous analysed period) whereas Canada (1016; 3.3%; 62.5) retained its share. The five most prolific journals were *Free Radical Biology and Medicine* (1017; 3.3%; 103.9), *Journal of Agricultural and Food Chemistry* (510; 1.7%; 122.7), *FASEB Journal* (470; 1.5%; 32.4), *Free Radical Research* (354; 1.2%; 53.6), and *Biochemical Pharmacology* (324; 1.1%; 63.9).  Figure S2 shows the citation network of the most prolific journals, apparently centred on food science, nutrition, and medicine journals.

**Figure 3:**
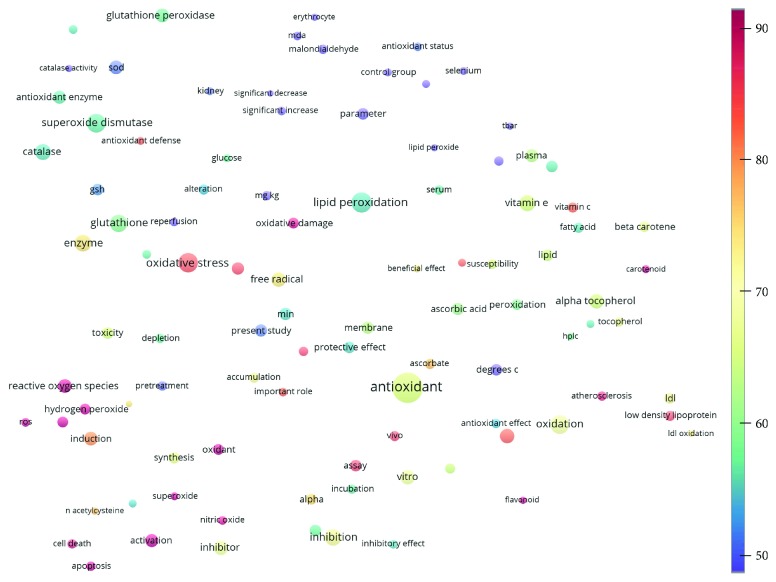
Term map for 1991–2000. The bubble map visualizes 99 terms (excluding the top 5000 common words from the Corpus of Contemporary American English) that appeared in at least 500 of the included manuscripts published during 1991–2000. Bubble size indicates the frequency of occurrence of the words (multiple appearances in a single publication count as one). Bubble color indicates the averaged citation count received by publications containing the word in their titles or abstracts. Two bubbles are in closer proximity if the two words had more frequent cooccurrence.

Topics with high citations included cell death (965 publications; 87.1 citations per publication) and atherosclerosis (1062; 86.1) (Figure 3). Some notable highly cited chemical terms included flavonoid (623; 167.3), carotenoid (683; 112.3), hydrogen peroxide (1430; 89.4), nitric oxide (826; 88.1), vitamin C (1160; 82.4), *N*-acetylcysteine (639; 77.4), ascorbate (1171; 77.3), beta-carotene (1317; 69.1), vitamin E (2869; 65.5), and alpha-tocopherol (2335; 65.2) (Table 1).

### 3.4. 2001–2010

There were 95,627 publications. Compared to the 1990s, the WoS category of *biochemistry/molecular biology* has accounted for a smaller share of publications (16,554; 17.3% of 95,627). The four other major categories were *food science technology* (13,424; 14.0%), *pharmacology/pharmacy* (12,253; 12.8%), *nutrition/dietetics* (7214; 7.5%), and *chemistry, applied* (7030; 7.4%). *Chemistry, applied* has regained its share compared to the previous decade. Meanwhile, *polymer science* (878; 0.9%) continued to have a reduced share. The five most prolific countries/regions were USA (20,504 publications; 21.4% of 95,627; 57.9 citations per publication), China (7458; 7.8%; 32.5), Japan (6944; 7.3%; 37.5), India (6829; 7.1%; 30.8), and Italy (5860; 6.1%; 42.9). The five most prolific journals were *Journal of Agricultural and Food Chemistry* (2907; 3.0%; 67.0), *Free Radical Biology and Medicine* (1927; 2.0%; 50.1), *Food Chemistry* (1866; 2.0%; 70.9), *Free Radical Research* (1014; 1.1%; 24.8), and *Food and Chemical Toxicology* (750; 0.8%; 49.0).  Figure S3 shows the citation network of the most prolific journals. *Food Chemistry* began to become comparable with *Journal of Agricultural and Food Chemistry* with regard to publication count and citations per publication.

**Figure 4:**
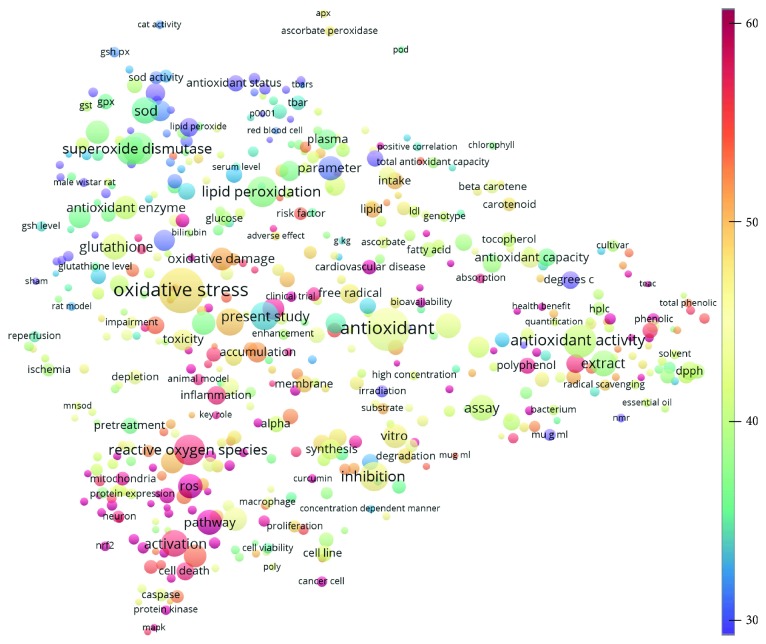
Term map for 2001–2010. The bubble map visualizes 503 terms (excluding the top 5000 common words from the Corpus of Contemporary American English) that appeared in at least 500 of the included publications published during 2001–2010. Bubble size indicates the frequency of occurrence of the words (multiple appearances in a single publication count as one). Bubble color indicates the averaged citation count received by publications containing the word in their titles or abstracts. Two bubbles are in closer proximity if the two words had more frequent cooccurrence.

Topics with high citations included nuclear factor erythroid 2-related factor 2 (Nrf2, 1126 publications; 100.7 citations per publication) (Figure 4), Alzheimer's disease (1477; 77.0), Parkinson's disease (977; 76.7), and cardiovascular disease (2305; 66.9). The most cited chemical terms related to antioxidants were curcumin (915; 75.4), flavonol (677; 70.5), phenolic acid (935; 64.4), anthocyanin (1560; 60.9), resveratrol (1159; 60.1), and kaempferol (635; 58.8) (Table 1). Interestingly, all of these are phytochemicals (plant secondary metabolites), as compared to the previous analysed periods, which have featured a higher share of studies focused on antioxidant vitamins and minerals.

### 3.5. 2011–2018

There were 169,269 publications. For the first time in history, *food science technology* (29,216; 17.3% of 169,269) has overtaken *biochemistry/molecular biology* (21,711; 12.8%) as the largest WoS category for antioxidant papers. *Pharmacology/pharmacy* (19,611; 11.6%) and *chemistry, applied* (12,346; 7.3%) had similar share relative to the 2000s. *Plant sciences* have an uprising trend (1.3% for 1990 and before, 3.3% for 1991–2000, 6.1% for 2001–2010, and 7.0% for 2011–2018) to take up the fifth spot in terms of the largest share of publications during 2011–2018. On the other hand, *polymer science*, once one of the five most prolific categories in 1990 and before, has maintained a small share in recent decades (9.9% for 1990 and before, 1.8% for 1991–2000, 0.9% for 2001–2010, and 1.6% for 2011–2018).

China (31,508 publications; 18.6% of 169,269; 8.5 citations per publication) has overtaken USA (19,997; 11.8%; 15.0) as the most prolific country. The rest of the five most prolific countries/regions were India (16,838; 9.9%; 7.5), Brazil (9322; 5.5%; 7.2), and South Korea (9069; 5.4%; 8.3). The five most prolific journals were *Food Chemistry* (3810; 2.3%; 17.2), *PLOS One* (2704; 1.6%; 12.5), *Journal of Agricultural and Food Chemistry* (2085; 1.2%; 15.1), *Molecules* (1646; 1.0%; 9.6), and *Free Radical Biology and Medicine* (1335; 0.8%; 18.7).  Figure S4 shows the citation network of the most prolific journals. *Food Chemistry* has finally overtaken *Journal of Agricultural and Food Chemistry* as the largest journal in terms of publication count of antioxidant papers. Also, notable is the rise of *PLOS One* and *Scientific Reports* (908; 0.5%; 4.2), two multidisciplinary journals that have published considerable number of antioxidant papers during this decade.

**Figure 5:**
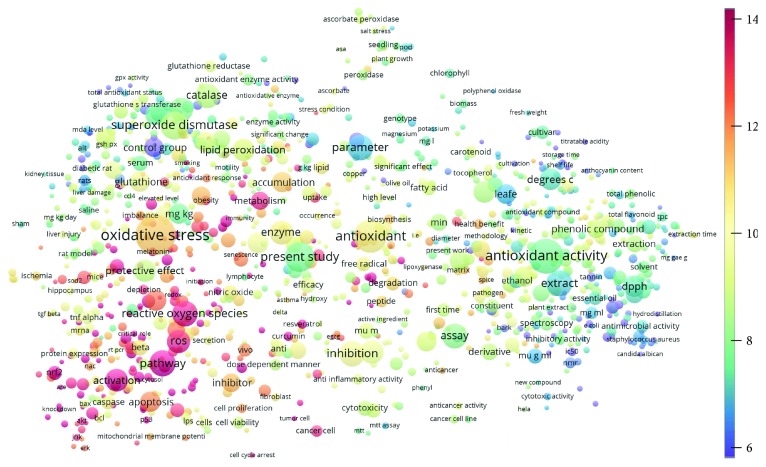
Term map for 2011–2018. The bubble map visualizes 1122 terms (excluding the top 5000 common words from the Corpus of Contemporary American English) that appeared in at least 500 of the included manuscripts published during 2011–2018. Bubble size indicates the frequency of occurrence of the words (multiple appearances in a single publication count as one). Bubble color indicates the averaged citation count received by publications containing the word in their titles or abstracts. Two bubbles are in closer proximity if the two words had more frequent cooccurrence.

Topics with high citations included autophagy (1091 publications; 18.3 citations per publication), Parkinson's disease (1466; 15.5), atherosclerosis (1958; 15.3), carcinogenesis (1086; 15.1), and Alzheimer's disease (2658; 15.1) (Figure 5). The most highly cited chemicals included curcumin (2131; 14.3), resveratrol (2141; 13.9), and cysteine (2410; 13.0) (Table 1). A previous literature analysis has reported that it takes about 3–7 years for scientific publications to reach the peak of citations per year during their citation life [16]. Therefore, it is reasonable to see that the general citations per publication during 2011–2018 were lower than those from the 2001–2010.

### 3.6. General Discussion

In overall, we have analysed nearly 300,000 publications on antioxidants that spanned over half a century. The results presented above have highlighted two polyphenols, curcumin and resveratrol, with high citations in the recent two decades. The potential health benefits of dietary curcumin and resveratrol have been discussed by the literature with a relevance to the control of redox signalling, inflammation, and autophagy [17, 18] and thus treatment of cancers including leukaemia [19–22]. Besides, polyphenols have also been demonstrated to have neuroprotective effects and beneficial effects against cardiovascular disease, diabetes, and aging [2, 3, 5, 9, 23]. Dietary polyphenols, together with vitamin E, which had high citations before 2000, can exert protective effect against Alzheimer's and Parkinson's diseases by modulating the overproduction of reactive oxygen species (ROS) that give rise to oxidative stress which in turn damages the neurons [24, 25]. The increased publication share of *food science technology* journals, with a leading example being *Food Chemistry*, has reflected that the attention is shifting towards dietary antioxidants.

Manuscript related to the nuclear factor erythroid 2-related factor 2 (Nrf2) also attracted high citations. Nrf2 is a transcription factor essential for regulating homeostasis, cytoprotection, and innate immunity and plays an important role in activating the protective genes that are antioxidant and anti-inflammatory [26].

The antioxidant research field has witnessed the increased publication share from China, India, and South Korea since the 2000s. Similar to the recently analysed ethnopharmacology and natural products publication fields, the contributions from Asian countries have been large, which could be partially explained by the notion that antioxidant assays require relatively less sophisticated equipment so that emerging research powers can also contribute comparable research volumes as traditionally established top research countries [27, 28].

## 4. Conclusions

There were 299,602 publications identified and analysed. The majority of them (295,426) were published after 1991. Article to review ratio was 12.5 : 1. The publications were mainly related to the categories *biochemistry/molecular biology*, *food science technology*, and *pharmacology/pharmacy*. Brazil, China, India, and South Korea have emerged as upcoming major contributors to antioxidant research besides USA, which has been dominating the field for half a century. Most prolific journals were *Food Chemistry*, *Journal of Agricultural and Food Chemistry*, *Free Radical Biology and Medicine*, and *PLOS One*. Clinical conditions with high citations included Alzheimer's disease, cancer, cardiovascular disease, and Parkinson's disease. Chemicals with high citations included alpha-tocopherol, anthocyanin, ascorbate, beta-carotene, carotenoid, curcumin, cysteine, flavonoid, flavonol, hydrogen peroxide, kaempferol, *N*-acetylcysteine, nitric oxide, phenolic acid, uric acid, vitamin C, vitamin E, selenium, and resveratrol (Table 1). In respect to antioxidant chemicals, a transition of the scientific interest has been observed, from research focused on antioxidant vitamins and minerals into more research attention focused on antioxidant phytochemicals (plant secondary metabolites).

## Figures and Tables

**Table 1:** Most cited antioxidants in the last several decades.

Time period	Chemical structure and name	Chemical term (number of publications; citations per publication)
1990 and before	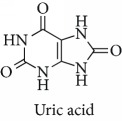	Uric acid (12; 212.1)
	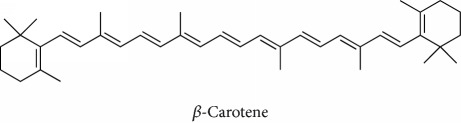	Beta-carotene (12; 189.3)
	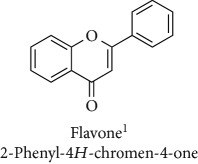	Flavonoid (15; 176.5)
	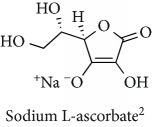	Ascorbate (14; 175.7)
	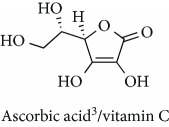	Vitamin C (22; 69.8)
	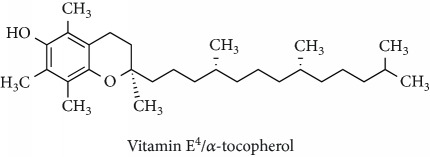	Vitamin E (121; 62.7)
	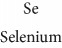	Selenium (35; 56.3)

1991–2000	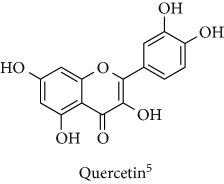	Flavonoid (623; 167.3)
	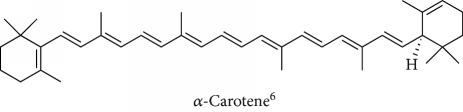	Carotenoid (683; 112.3)
	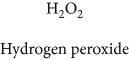	Hydrogen peroxide (1430; 89.4)
	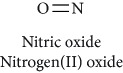	Nitric oxide (826; 88.1)
	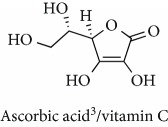	Vitamin C (1160; 82.4)
	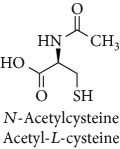	*N*-Acetylcysteine (639; 77.4)
	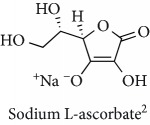	Ascorbate (1171; 77.3)
	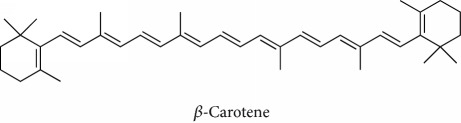	Beta-carotene (1317; 69.1)
	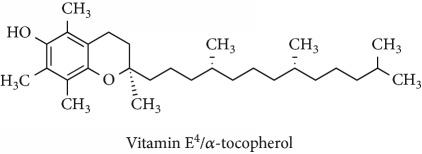	Vitamin E (2869; 65.5) Alpha-tocopherol (2335; 65.2)

2001–2010	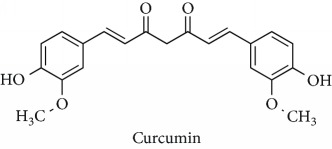	Curcumin (915; 75.4)
	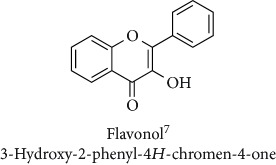	Flavonol (677; 70.5)
	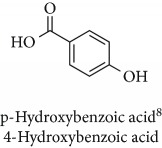	Phenolic acid (935; 64.4)
	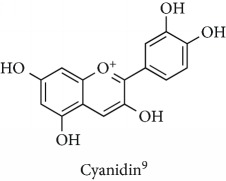	Anthocyanin (1560; 60.9)
	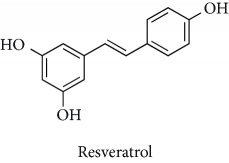	Resveratrol (1159; 60.1)
	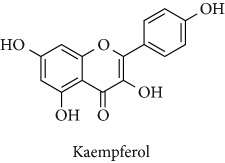	Kaempferol (635; 58.8)

2011–2018	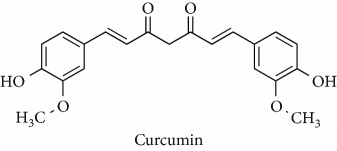	Curcumin (2131; 14.3)
	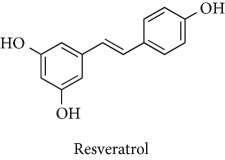	Resveratrol (2141; 13.9)
	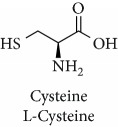	Cysteine (2410; 13.0)

^1^Flavone backbone of flavonoids. ^2^The most common ascorbate. ^3^Ascorbic acid is one form “vitamer” of vitamin C. ^4^ *α*-Tocopherol is the most active form of vitamin E (includes also other tocopherols and tocotrienols). ^5^An example as the most common flavonoid. ^6^It is the second most common form of carotene. ^7^Backbone of a flavonol. ^8^An example as the most common phenolic acid. ^9^The most common frequently occurring in the nature anthocyanin.

## Supplementary Materials

Supplementary Materials Data File S1: supplementary data sheets of all the terms remained after the exclusion of the 5000 common words and their citations per publication, per each of the four survey periods. Figure S1: journal citation network for 1990 or before. The bubble map visualizes 90 journals that published at least 10 of the included publications published in 1990 or before. Bubble size indicates the number of publications of the journal. Bubble color indicates the averaged citation count received by publications of the journal. Two bubbles are in closer proximity if the two journals cited each other more frequently. The lines indicate the 500 strongest citation links. Figure S2: journal citation network for 1991–2000. The bubble map visualizes 45 journals that published at least 100 of the included publications published during 1991–2000. Bubble size indicates the number of publications of the journal. Bubble color indicates the averaged citation count received by publications of the journal. Two bubbles are in closer proximity if the two journals cited each other more frequently. The lines indicate the 500 strongest citation links. Figure S3: journal citation network for 2001–2010. The bubble map visualizes 190 journals that published at least 100 of the included publications published during 2001–2010. Bubble size indicates the number of publications of the journal. Bubble color indicates the averaged citation count received by publications of the journal. Two bubbles are in closer proximity if the two journals cited each other more frequently. The lines indicate the 500 strongest citation links. Figure S4: journal citation network for 2011–2018. The bubble map visualizes 327 journals that published at least 100 of the included publications published during 2001–2010. Bubble size indicates the number of publications of the journal. Bubble color indicates the averaged citation count received by publications of the journal. Two bubbles are in closer proximity if the two journals cited each other more frequently. The lines indicate the 500 strongest citation links.
