# Supplementary figures and images for: Antioxidants: Scientific Literature Landscape Analysis
Source: Oxid Med Cell Longev. 2019 Jan 8;2019:8278454. doi: 10.1155/2019/8278454 (PMC6341248; doi:10.1155/2019/8278454)

**Figure S1.**

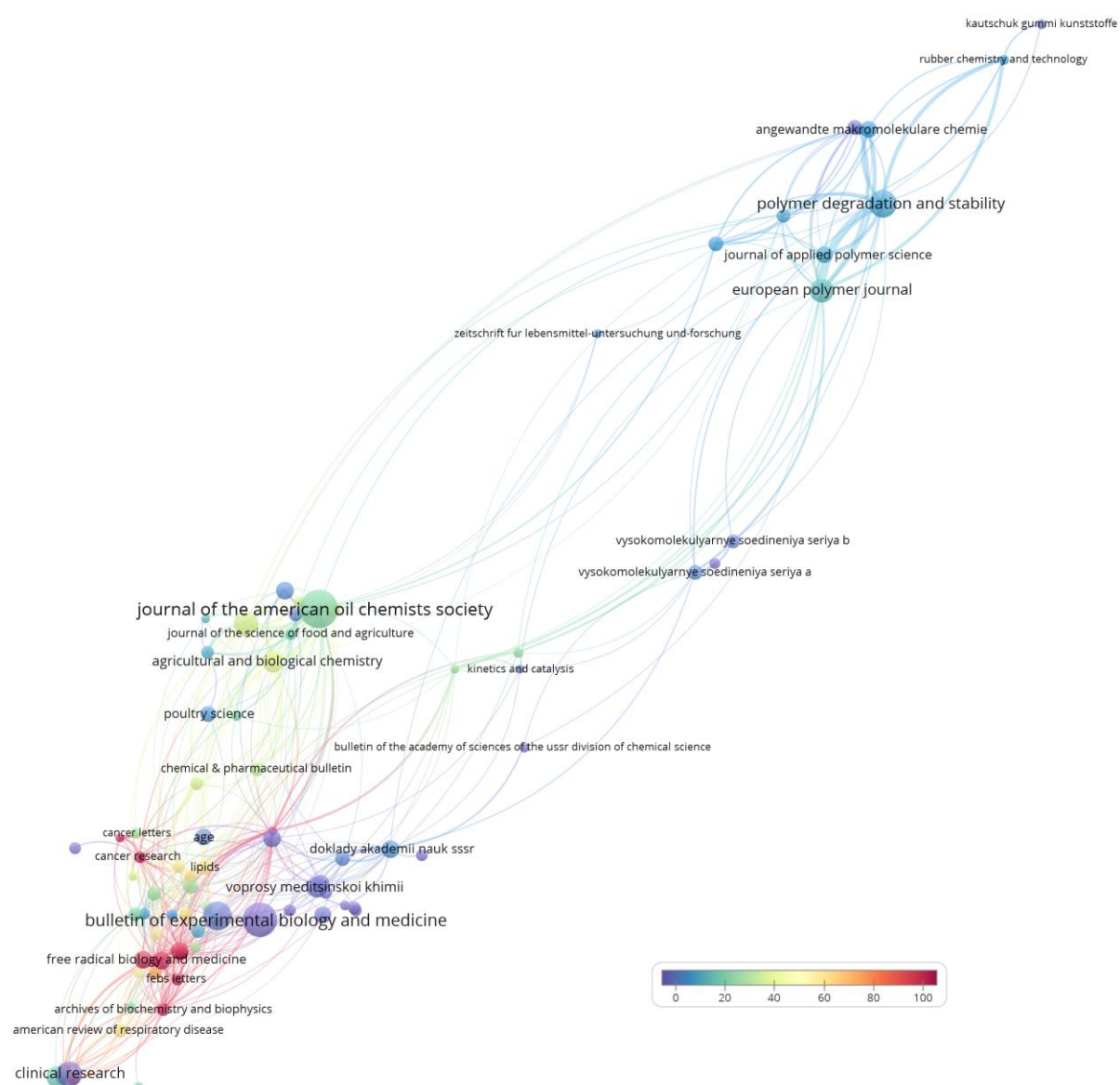

**Figure S2.**

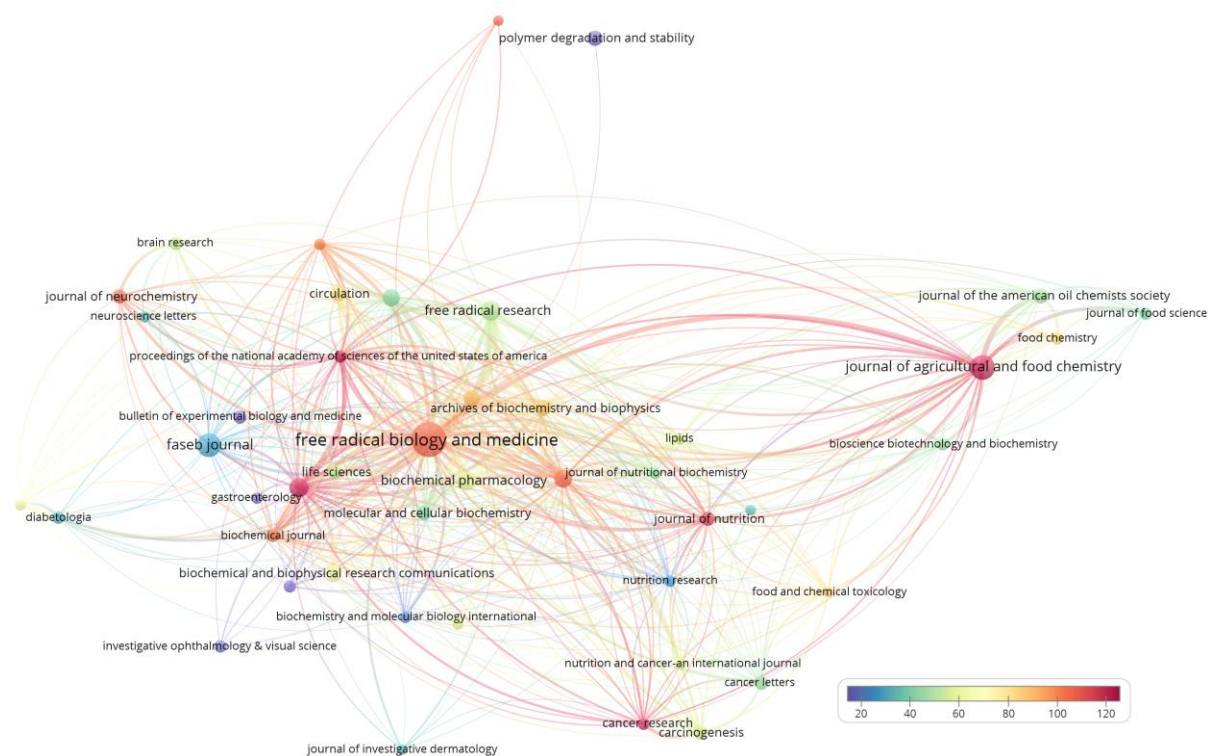

**Figure S3.**

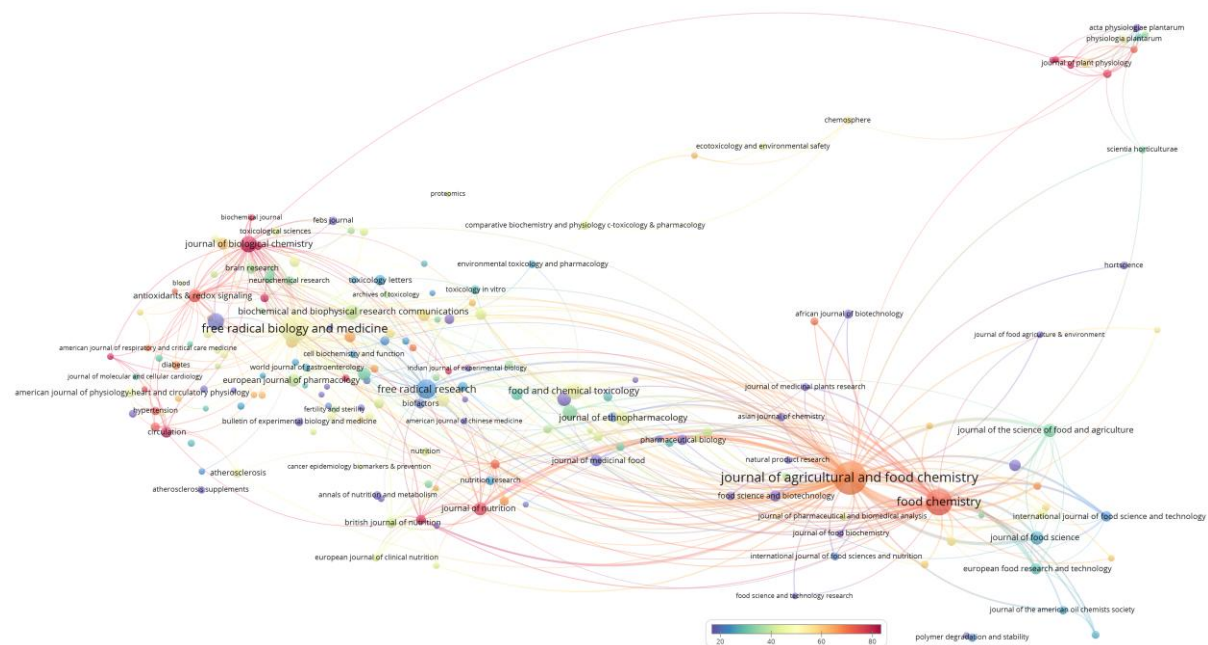

**Figure S4.**

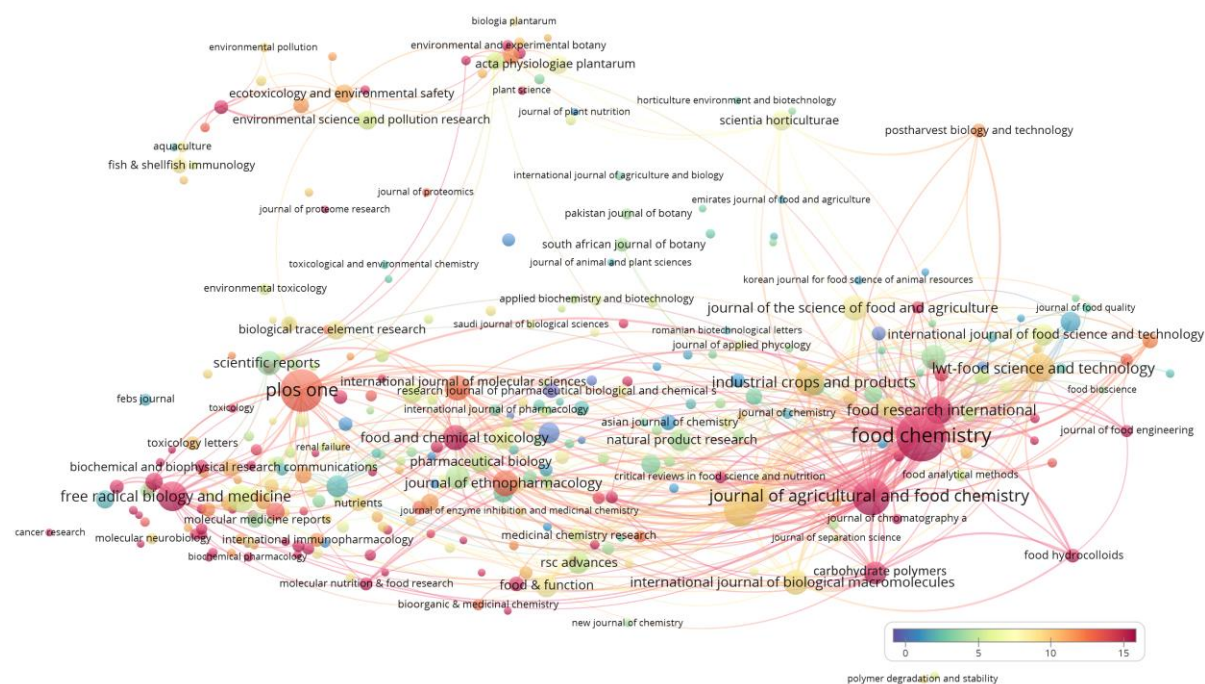

Supplement: Supplementary Materials — Data File S1: supplementary data sheets of all the terms remained after the exclusion of the 5000 common words and their citations per publication, per each of the four survey periods. Figure S1: journal citation network for 1990 or before. The bubble map visualizes 90 journals that published at least 10 of the included publications published in 1990 or before. Bubble size indicates the number of publications of the journal. Bubble color indicates the averaged citation count received by publications of the journal. Two bubbles are in closer proximity if the two journals cited each other more frequently. The lines indicate the 500 strongest citation links. Figure S2: journal citation network for 1991–2000. The bubble map visualizes 45 journals that published at least 100 of the included publications published during 1991–2000. Bubble size indicates the number of publications of the journal. Bubble color indicates the averaged citation count received by publications of the journal. Two bubbles are in closer proximity if the two journals cited each other more frequently. The lines indicate the 500 strongest citation links. Figure S3: journal citation network for 2001–2010. The bubble map visualizes 190 journals that published at least 100 of the included publications published during 2001–2010. Bubble size indicates the number of publications of the journal. Bubble color indicates the averaged citation count received by publications of the journal. Two bubbles are in closer proximity if the two journals cited each other more frequently. The lines indicate the 500 strongest citation links. Figure S4: journal citation network for 2011–2018. The bubble map visualizes 327 journals that published at least 100 of the included publications published during 2001–2010. Bubble size indicates the number of publications of the journal. Bubble color indicates the averaged citation count received by publications of the journal. Two bubbles are in closer proximity if the two journals [file 8278454.f1.zip › Supplementary Figures.pdf]
